# Assessment of Cardiovascular Disease (CVD) Risk Awareness Among Individuals With Type 2 Diabetes at Qassim University Medical City

**DOI:** 10.7759/cureus.79301

**Published:** 2025-02-19

**Authors:** Eman A Alotaibi, Ghaday S Almutairi, Rasha H Almutairi, Nuha M Alzaydi, Mariyyah M Alharbi, Shikhah G Alharbi, Raghad Alwehaibi, Tanveer N Khan, Bader H Alharbi

**Affiliations:** 1 Family and Community Medicine, College of Medicine, Qassim University, Buraydah, SAU; 2 College of Medicine, Qassim University, Buraydah, SAU; 3 Medicine and Surgery, Batterjee Medical College, Jeddah, SAU

**Keywords:** cardiovascular, diabetes, health education, knowledge, risk awareness

## Abstract

Introduction: Type 2 diabetes (T2D), marked by high blood sugar levels, significantly impacts bodily systems and can result in various health complications. In Saudi Arabia, it poses a widespread health concern, particularly in urban areas. The condition is linked to both small and large blood vessel complications, including issues such as eye disease, kidney damage, and heart-related problems. Factors like gender, location, and associated small blood vessel conditions can affect the occurrence of cardiovascular diseases (CVD). Strengthening collaboration with healthcare professionals is crucial to reducing heart disease cases and improving comprehension of diabetes-related consequences.

Methods: This was a descriptive cross-sectional study among patients with T2D at the outpatient clinics of Qassim University Medical City in Saudi Arabia. The study utilized a self-administered questionnaire which was sent to patients attending outpatient clinics through a structured paper-based survey. The questionnaire has a socio-demographic section and a 25-item assessment to evaluate the knowledge of diabetic patients concerning cardiovascular disease risk.

Results: Of the 153 diabetic patients, 85 (55.6%) were males, and 39 (25.5%) were between 40 and 49 years old. A family history of diabetes has been reported by 116 (75.8%) of the patients. The overall mean knowledge score was 14.8 out of 25 points. 48 (31.4%) were considered to have good knowledge; however, more than one-third of the diabetes mellitus (DM) patients (N=52; 34%) had poor knowledge of CVD risk. Increased knowledge was seen more frequently in the older age group, the male gender, and those with longer DM durations.

Conclusion: The patients with T2D had an inadequate understanding of cardiovascular disease risk. Nevertheless, older male patients with an extended period of diabetes mellitus exhibited a superior understanding of CVD risk. Additional longitudinal studies are necessary to determine the variables and the extent of information regarding CVD risk among diabetic individuals in our region.

## Introduction

Type 2 diabetes (T2D) is a chronic condition characterized by elevated blood glucose levels, which can significantly impact various biological systems, leading to severe health complications [1]. Recent studies have highlighted a significant rise in T2D cases across the Gulf Cooperation Council (GCC) countries, which include Saudi Arabia, Bahrain, Qatar, Oman, Kuwait, and the United Arab Emirates. The prevalence rates in these nations range from 8% to 22%, as reported by the International Diabetes Federation (IDF) [2]. T2D is associated with numerous microvascular and macrovascular complications, including cardiovascular disease (CVD), diabetic retinopathy, and nephropathy [3]. Among these, CVD is the leading cause of mortality in individuals with T2D, despite being largely preventable through proper management [3,4]. The risk of a cardiovascular event in T2D patients without a prior history of CVD is influenced by several factors, such as gender, geographic region, and the presence of microvascular complications [5].

A global study, the "Taking Diabetes to Heart" survey, collected data from 12,695 individuals across 133 countries. The findings revealed that 44% of participants had been living with diabetes for over nine years, yet only 17% considered themselves at high risk for CVD, despite most having at least one CVD risk factor [6]. Furthermore, 28% of respondents reported experiencing one or more CVD events, 17% had never discussed their CVD risk with a healthcare provider, and 9% were unaware of CVD and its associated risk factors [6]. These results highlight the critical need for healthcare professionals to enhance awareness and address the growing burden of CVD in individuals with T2D.

Although studies from various regions have explored the awareness of CVD risks among T2D patients, significant gaps remain in certain populations. For instance, research in Sweden revealed that limited knowledge of CVD risks improved as participants gained a deeper understanding of T2D’s severity, emphasizing the importance of patient education [7]. Similarly, studies from the USA, Ethiopia, Nigeria, and Saudi Arabia underscored the necessity of tailored educational strategies to enhance patient awareness and promote better self-management practices [8-13].

Despite the growing prevalence of T2D and its complications globally, there is a notable lack of research examining CVD risk awareness among adults with T2D in the Qassim region of Saudi Arabia. This study aims to fill this gap by conducting a comprehensive cross-sectional investigation into the knowledge, perceptions, and behaviors related to CVD risk among T2D patients in Qassim. By improving understanding and awareness, this research seeks to develop strategies for better diabetes management and the prevention of cardiovascular complications.

## Materials and methods

Study design and population

The study employed a cross-sectional approach, focusing on adults diagnosed with T2D who regularly visited the diabetes clinic at Qassim University Medical City for follow-up care. Participants included both men and women actively managing their condition. The exclusion criteria included non-diabetic persons. This study included adults diagnosed with T2D attending outpatient clinics at Qassim University Medical City. Participants were recruited from various age groups, with the inclusion criteria allowing for individuals aged 30 years and above. The majority of participants fell within the 40-49 age group (25.5%) and 50-59 age group (23.5%), ensuring representation across middle-aged and older adults. The questionnaire was translated into Arabic, the native language of all participants, to ensure comprehension.

Sampling and sample size

The Epi Info program version 7.2.5.0 (CDC, Atlanta, GA, USA) was utilized to calculate the sample size, employing a 95% confidence interval (CI), a 5% margin of error (MOE), and an expected frequency of 50%. The anticipated sample size was 132 individuals. Convenience was utilized to enroll eligible patients who met the inclusion criteria.

Data collection tools

Data were gathered via a pre-validated, self-administered questionnaire, modified from other studies and translated into Arabic [12]. The questionnaire sought to evaluate awareness of CVD risks in persons with T2D. It comprised three sections: socio-demographic characteristics, diabetes-related factors, and the Heart Disease Fact Questionnaire (HDFQ) [14]. The last segment employed an Arabic-translated variant of the HDFQ to evaluate participants' comprehension of CVD risks. A pilot research with seven individuals was executed to assess the clarity, feasibility, and appropriateness of the questionnaire, with these people subsequently eliminated from the final sample. Revisions were implemented to enhance clarity, and the finalized version of the questionnaire was conducted as a paper-based survey.

Measure

The assessment of CVD knowledge was conducted using a 25-item questionnaire. Participants replied with "yes" (given a score of 1) or "no/I don’t know" (assigned a score of 0). The cumulative score varied from 0 to 25, with increasing numbers indicating increased expertise. Knowledge levels were classified as inadequate (<50%), moderate (50-75%), or proficient (>75%) [15].

Data analysis

The data underwent entry and cleaning processes in Microsoft Excel (Redmond, WA, USA), where duplicate entries, inconsistencies, and incomplete responses were identified and rectified. The refined dataset was then transferred to SPSS software (version 26; IBM Corp., Armonk, NY, USA). Descriptive statistics were summarized using frequencies and percentages. Kolmogorov-Smirnov test and Mann-Whitney Z-test were conducted to explore associations between patient knowledge and socio-demographic factors. A P-value < 0.05 was deemed statistically significant.

Ethical considerations

The research obtained ethical clearance from the Qassim University Research Ethics Committee (No. 24-96-01). Prior to participation, all individuals were provided with a detailed explanation of the study objectives, procedures, and potential risks and benefits. Informed consent was obtained in Arabic, the native language of all participants, to ensure clear understanding. For individuals with limited literacy skills, trained research staff assisted by verbally explaining the consent form and addressing any questions. Participation was voluntary, with no financial or alternative incentives provided. Confidentiality was rigorously upheld, with data utilized exclusively for research reasons. The research was free from conflicts of interest and did not receive financing from external entities.

## Results

This study recruited 153 diabetes mellitus (DM) patients from 132 calculated sample sizes (response rate: 115%) (Table 1). Thirty-nine (25.5%) were between 40 - 49 years old. More than half were males (N=85; 55%), and nearly half (N=72; 47.1%) were bachelor's degree holders. Patients who were married constituted 96 (62.7%). With regards to monthly income, 37 (24.2%) were earning less than 5,000 SAR per month. Nearly all patients (N=143; 93.5%) lived in urban, mostly in villas (N=79; 51.6%). Also, 48 (31.4%) had less than five years DM duration. In addition, 116 (75.6%) reported to have family history of diabetes.

**Table 1:** Socio-demographic characteristics of the diabetes mellitus (DM) patients (n=153)

Study variables	N (%)
Age group in Years	
<30	21 (13.7%)
30 – 39	19 (12.4%)
40 – 49	39 (25.5%)
50 – 59	36 (23.5%)
60 – 69	28 (18.3%)
≥70	10 (06.5%)
Gender	
Male	85 (55.6%)
Female	68 (44.4%)
Educational level	
Uneducated	09 (05.9%)
Can read and write	03 (02.0%)
Primary school	07 (04.6%)
Intermediate education	11 (07.2%)
Secondary education	23 (15.0%)
Bachelor's degree	72 (47.1%)
Postgraduate education	28 (18.3%)
Marital status	
Single	25 (16.3%)
Married	96 (62.7%)
Divorced	21 (13.7%)
Widowed	11 (07.2%)
Gross Monthly Income (SAR)	
<5,000	37 (24.2%)
5,000 - 8,000	32 (20.9%)
8,001 - 10,000	16 (10.5%)
10,001 - 15,000	18 (11.8%)
15,001 - 20,000	23 (15.0%)
>20,000	27 (17.6%)
Place of residence	
Urban	143 (93.5%)
Rural	10 (06.5%)
Housing	
Villa	79 (51.6%)
Floor	26 (17.0%)
Apartment	27 (17.6%)
Traditional house	21 (13.7%)
Duration of diabetes in years	
<5	48 (31.4%)
5 - 10	39 (25.5%)
11 - 15	25 (16.3%)
16 - 20	16 (10.5%)
>20	25 (16.3%)
Family history of diabetes	
Yes	116 (75.8%)
No	37 (24.2%)

**Figure 1:**
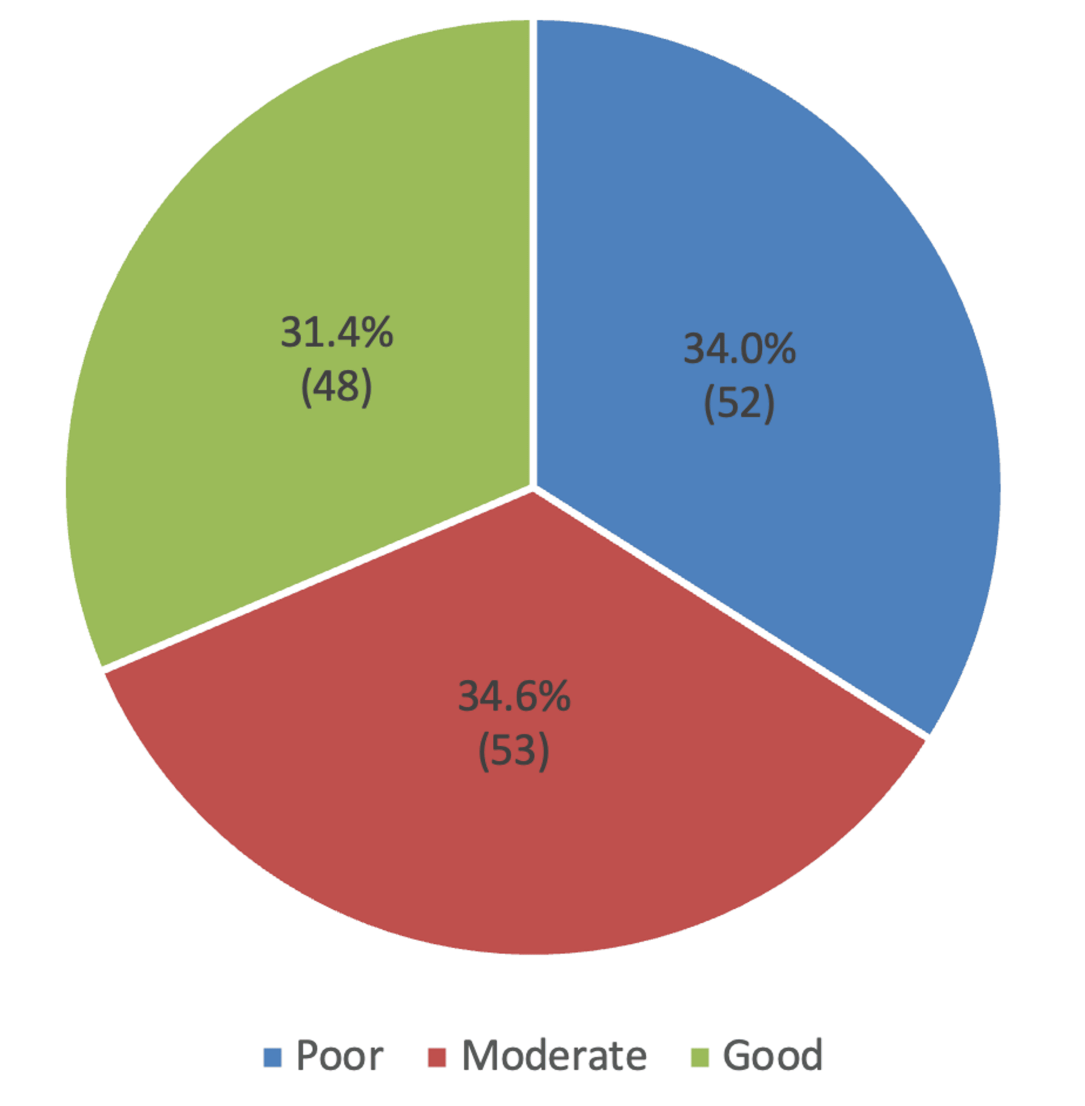
Level of knowledge of cardiovascular disease (CVD) risk

Table 2 below shows that the top five knowledge items showing the highest ratings were "A person who stops smoking will lower their risk of developing heart disease" (N=113; 73.9%), "High blood pressure is a risk factor for heart disease" (N=113; 73.9%), "Smoking is a risk factor for heart disease" (N=112; 73.2%), "Regular physical activity will lower a person's chance of getting heart disease" (N=111; 72.5%), and "Person who has diabetes can reduce their risk of developing CVD if they keep their weight under control" (N=109; 71.2%), while knowledge items "People with diabetes rarely have high cholesterol" (N=56; 36.6%), "If your 'good' cholesterol (HDL) is high, you are at risk for CVD" (N=57; 37.3%), and "Men with diabetes have a higher risk of CVD than women with diabetes" (N=59; 38.6%) were the top three with the lowest ratings. The overall mean knowledge score was 14.8 (SD 6.29). Consequently, the degrees of knowledge were categorized as poor, moderate, and good, comprising 52 (34%), 53 (34.6%), and 48 (31.4%), respectively (Figure 1).

**Table 2:** Assessment of the knowledge of cardiovascular disease (CVD) (n=153)

Knowledge items	Yes (%)
A person who quits smoking will reduce their risk of developing CVD.	113 (73.9%)
Hypertension is a risk factor for CVD	113 (73.9%)
Smoking constitutes a risk factor for CVD	112 (73.2%)
Consistent physical activity will diminish an individual's likelihood of getting CVD.	111 (72.5%)
Individuals with diabetes can mitigate their risk of getting CVD by maintaining weight management.	109 (71.2%)
Elevated cholesterol levels constitute a risk factor for the onset of CVD	107 (69.9%)
As a person ages, their risk of acquiring CVD increases.	105 (68.6%)
Elevated levels of low-density lipoprotein (LDL) cholesterol augment the risk of CVD	104 (68.0%)
A diabetic individual can mitigate their risk of CVD by maintaining controlled blood sugar levels.	103 (67.3%)
Excess weight elevates an individual's risk for CVD.	101 (66.0%)
Walking and gardening are seen as activities that can reduce an individual's risk of acquiring CVD.	99 (64.7%)
Maintaining cholesterol levels within a healthy range can reduce the risk of CVD in those with diabetes.	99 (64.7%)
Individuals with diabetes can mitigate their risk of CVD by maintaining controlled blood pressure.	99 (64.7%)
Prolonged elevated blood sugar levels can elevate cholesterol levels, hence increasing the risk of CVD.	93 (60.8%)
Diabetes constitutes a risk factor of CVD.	91 (59.5%)
Elevated blood glucose levels exert pressure on the heart.	91 (59.5%)
A familial predisposition to CVD increases your likelihood of having the condition.	86 (56.2%)
A person is constantly aware when they have heart illness.	85 (55.6%)
Individuals with diabetes often exhibit reduced levels of HDL cholesterol.	76 (49.7%)
Exclusively engaging in gym workouts or exercise classes will reduce an individual's risk of CVD.	68 (44.4%)
Consumption of fatty foods does not influence blood cholesterol levels.	65 (42.5%)
Maintaining blood pressure will diminish the likelihood of getting CVD.	59 (38.6%)
Men with diabetes exhibit a higher risk of cardiovascular illness compared to women with diabetes	59 (38.6%)
Elevated levels of 'good' cholesterol (HDL) increase your risk of CVD.	57 (37.3%)
Individuals with diabetes infrequently exhibit elevated cholesterol levels.	56 (36.6%)
Total knowledge score (mean ± SD)	14.8 ± 6.29
Level of knowledge	
Poor	52 (34.0%)
Moderate	53 (34.6%)
Good	48 (31.4%)

Investigating the correlation between knowledge and the socio-demographic attributes of patients (Table 3), we observed that elevated knowledge scores correlated with advanced age (Z=3.184; p=0.001), male sex (Z=2.402; p=0.016), and an extended duration of diabetes (Z=2.069; p=0.069). No notable variations were detected in the knowledge score concerning education, marital status, gross monthly income, place of living, housing, and hereditary diabetic predisposition (all p>0.05).

**Table 3:** Association between knowledge and the socio-demographic attributes of patients (n=153) § Mann-Whitney Z-test used. ** Significant at p<0.05 level.

Factor	Knowledge Score (25) Mean ± SD	Z-test	P-value ^§^
Age group			
<50 years	13.1 ± 6.81	3.184	0.001 **
≥50 years	16.6 ± 5.16		
Gender			
Male	16.1 ± 5.87	2.402	0.016 **
Female	13.2 ± 6.47		
Educational level			
Secondary or below	14.8 ± 5.36	0.131	0.896
Bachelor's degree or higher	14.8 ± 6.76		
Marital status			
Unmarried	14.3 ± 7.33	0.503	0.615
Married	15.1 ± 5.61		
Gross Monthly Income (SAR)			
≤10,000	14.7 ± 6.37	0.000	1.000
>10,000	14.9 ± 6.23		
Place of residence			
Urban	14.7 ± 6.19	0.470	0.639
Rural	15.2 ± 8.01		
Housing			
Villa	14.7 ± 6.01	0.022	0.982
Non-villa	14.9 ± 6.62		
Duration of diabetes			
≤10 years	13.7 ± 6.85	2.069	0.039 **
>10 years	16.2 ± 5.17		
Family history of diabetes			
Yes	14.4 ± 6.69	1.071	0.284
No	15.9 ± 4.72		

## Discussion

This study investigated the awareness of T2D patients regarding CVD risk. This study's findings may substantially enhance the literature, given the strong correlation between diabetes and CVD. This study would also provide more awareness about the need for health education among diabetic patients and the importance of enhancing their diabetes self-care knowledge. Improving diabetes self-management could lead to reduced rates of diabetes complications and enhance overall patient outcomes.

The understanding of CVD risk among diabetic people was inadequate. Our results suggest that even though 31.4% (N=48) of our population showed good knowledge, over one-third of the patients exhibited inadequate knowledge (mean score: 14.8 out of 25 points). This aligns with the previous report of Obirikorang et al. (2016), indicating that 60% of diabetic patients were unaware of diabetes complications, and 26.9% possessed insufficient information [11]. Supporting these reports, a qualitative study by Jutterström et al. (2023) narrated a lack of understanding of DM patients regarding their disease. Patients appeared to lack a comprehensive understanding of their heightened cardiovascular disease risk or failed to recognize that their self-management efforts were intended to mitigate this risk [7]. In contrast, several studies documented a much better knowledge of CVD risk among the diabetic population [8,10,12,13,16-18]. These variations could be due to the study design, regional settings, and research focus. Our study emphasized the importance of diabetes health education. Healthcare providers should ensure that diabetic patients are adequately informed about the various diabetic complications and how to manage their disease. Education from healthcare practitioners can markedly enhance quality of life and reduce the risk of diabetic complications, including CVD.

Our findings suggest that age group was identified as a predictor affecting knowledge, wherein increased knowledge was associated with the older age groups (p=0.001). Consistent with our result, various studies documented that age was positively associated with knowledge [9,12,16]. Further, our results indicate that the male gender was associated with better knowledge than their female counterparts (p=0.016), and patients with longer DM duration were also associated with an increased understanding of the CVD risk. This contradicted the reports of Adeyemi et al. (2018). The study indicated that female patients demonstrated better knowledge of DM retinopathy, whereas male patients were seen to have more knowledge of erectile dysfunction [10]. However, studies conducted by Tovar and Clark (2013) and Obirikorang et al. (2016) as well as Workina et al. (2022) showed that education and urban residence were strong predictors of knowledge [8,11,18]. In our study, however, educational level, marital status, gross monthly income, place of residence, housing, and family history of diabetes were irrelevant factors to the knowledge (p>0.05), which may need further investigations. These differences could be attributed to several reasons, including population diversity, cultural and behavioral factors, and access to healthcare.

The gaps in understanding CVD risk stemmed from the specific details used to assess the knowledge. For instance. Although many patients recognized the risk factors for cardiovascular disease, including smoking, hypertension, hyperlipidemia, advanced age, and high bad cholesterol, their knowledge about high cholesterol adverse effects among the DM population was poor. Also, their understanding of the risk of heart disease from having high 'good' cholesterol levels and the importance of keeping blood pressure and eating fatty foods affecting blood cholesterol levels were underappreciated as well. On the contrary, most patients knew that regular physical activity, weight control, and controlled blood sugar can reduce the risk of CVD. In alignment with our findings, diabetic individuals in Ghana exhibited atypical awareness regarding diabetes mellitus risk factors, including renal disease (5.4%), cardiovascular disease (9.2%), ocular illness (17.7%), and sexual dysfunction (21.5%). Only the knowledge about diabetic foot as a risk factor for DM complications showed adequate knowledge (51.5%) [11]. However, DM patients in Taif showed a better understanding of CVD risks, including smoking (79.5%), high blood pressure (76.9%), bad cholesterol (74.4%), and older age (66%). They also knew that keeping the blood sugar under control and regular exercise reduces the risk of heart disease [17]. The lack of understanding of these risk factors could increase morbidity and mortality risk among this population group. Hence, continuous health education is critical to enhance DM self-care, reduce the risk of CVD, and improve patients' quality of life.

Since this study provides more light on patients with T2D awareness of their risk for cardiovascular disease, certain limitations should be noted. The study's cross-sectional design restricts our capacity to infer causality, and the mostly urban sample could not accurately represent the experiences of people in rural regions. Furthermore, even though the sample size exceeded the estimated need, growing the cohort even more could improve the results' generalizability. These elements must be taken into account while analyzing the findings and planning further studies.

To improve knowledge about diabetes and CVD among patients, patient-centered education programs should include structured sessions in clinics, simplified educational materials, and mobile health applications to enhance awareness. Community-based awareness campaigns using social media, television, and local outreach efforts can help spread information, while free screening camps can provide early detection and counseling.

## Conclusions

The knowledge of the patients with T2D regarding cardiovascular disease risk was unfavorable. Younger age groups, females, and shorter DM duration were seen as influential factors affecting patients' knowledge of CVD risk. There is a need to improve the knowledge of DM patients regarding CVD risk. A good understanding of the disease is crucial due to their strong association. Further, health education given by healthcare providers is also critical to learning more about managing their disease. Improving cardiovascular risk management in DM patients could reduce the burden of CVD and enhance life expectancy and quality of life. This method encompasses the broader goal of decreasing the overall complications of diabetes, warranting better health outcomes.
